# Enhanced aluminum tolerance in sugarcane: evaluation of *SbMATE* overexpression and genome-wide identification of *ALMT*s in *Saccharum* spp.

**DOI:** 10.1186/s12870-021-02975-x

**Published:** 2021-06-29

**Authors:** Ana Paula Ribeiro, Felipe Vinecky, Karoline Estefani Duarte, Thaís Ribeiro Santiago, Raphael Augusto das Chagas Noqueli Casari, Aline Forgatti Hell, Bárbara Andrade Dias Brito da Cunha, Polyana Kelly Martins, Danilo da Cruz Centeno, Patricia Abrão de Oliveira Molinari, Geraldo Magela de Almeida Cançado, Jurandir Vieira de Magalhães, Adilson Kenji Kobayashi, Wagner Rodrigo de Souza, Hugo Bruno Correa Molinari

**Affiliations:** 1 Genetics and Biotechnology Laboratory, Embrapa Agroenergy, Brasilia, 70770-901 DF Brazil; 2 Centre of Natural Sciences and Humanities, Federal University of ABC, São Bernardo do Campo, SP 09606-045 Brazil; 3 Phytopathology Department, University of Brasília, Brasília, Distrito Federal 70910-900 Brazil; 4 Center of Genetic Engineering and Molecular Biology, Embrapa GenClima, AC Unicamp University City, Campinas, 13083-886 SP Brazil; 5 Applied Biology Center (NBA), Embrapa Maize and Sorghum, Sete Lagoas, 35701-970 MG Brazil

**Keywords:** Aluminum, Sugarcane, *MATE*, *ALMT*, Abiotic stress, Hydroponic system

## Abstract

**Background:**

A major limiting factor for plant growth is the aluminum (Al) toxicity in acidic soils, especially in tropical regions. The exclusion of Al from the root apex through root exudation of organic acids such as malate and citrate is one of the most ubiquitous tolerance mechanisms in the plant kingdom. Two families of anion channels that confer Al tolerance are well described in the literature, ALMT and MATE family.

**Results:**

In this study, sugarcane plants constitutively overexpressing the *Sorghum bicolor MATE* gene (*SbMATE*) showed improved tolerance to Al when compared to non-transgenic (NT) plants, characterized by sustained root growth and exclusion of aluminum from the root apex based on the result obtained with hematoxylin staining. In addition, genome-wide analysis of the recently released sugarcane genome identified 11 *ALMT* genes and molecular studies showed potential new targets for aluminum tolerance.

**Conclusions:**

Our results indicate that the transgenic plants overexpressing the *Sorghum bicolor MATE* has an improved tolerance to Al. The expression profile of ALMT genes revels potential candidate genes to be used has an alternative for agricultural expansion in Brazil and other areas with aluminum toxicity in poor and acid soils.

**Supplementary Information:**

The online version contains supplementary material available at 10.1186/s12870-021-02975-x.

## Background

Sugarcane represents a very important economic crop worldwide, and its production is intended mainly for ethanol and sugar production. Furthermore, the biomass released from the sugarcane process can be used as lignocellulosic material to be degraded by microorganisms to generate renewable fuels and added-value products. Despite its great importance, sugarcane plantation areas are decreasing, especially in Brazil, the largest sugarcane producer [1, 2]. In Brazil, for example, an alternative for the expansion of sugarcane planting area is the *Cerrado* region, a biome characterized by acidic soil. Therefore, a great effort has been made by the scientific community to improve traits for sugarcane growth in poor and acid soils [3].

At low pH conditions, aluminum (Al), a natural constituent of the clay fraction of the soil, assumes ionic forms that are highly phytotoxic (Al^3+^ or Al(H_2_O)_6_^3+^), damaging the root system and restricting plant development [4–6] . The initial symptoms of Al toxicity in plants are the inhibition of root growth, changes in the root morphology as atrophy of root hair, as the top leaves show chlorosis and scorched symptom of Al toxicity. In addition, Al^3+^ can affect the division and elongation of root tip cells, causing irregular arrangement of cells and thickening of cell wall. Aluminum (Al^3+^) ions at micromolar concentrations and in a short time of exposure can cause severe toxic effects in plants [7–10].

Toxicity by Al decreases water absorption efficiency present in the subsoil, potentiating the effects of drought stress on reducing crop yields in acid soils. Crops sensitive to Al undergo severe reductions in grain and biomass production in acid soils. Liming is a common practice used to raise soil pH, however, besides the significant additional cost, it is not effective in the most common situation where toxic Al is in the deeper layers of the soil and the proper development of the root system for maintenance of plant production is compromised [11]. Thus, the use of genotypes adapted to acid soil conditions along with the addition of limestone and adequate fertilization are some of the strategies used in soils with high levels of Al^3+^ [2, 4].

The understanding of the Al-resistance mechanisms and the development of strategies to confer plant resistance have been investigated constantly in major crops, but the precise mechanism of Al tolerance and molecular mechanisms in plants are still not clear, mainly due to Al interference in multiple sites in apoplast and symplast, and the complexity of plant self-defense [8, 12, 13]. One of the most ubiquitous tolerance mechanisms in the plant kingdom is the exclusion of Al from the root apex through root exudation of organic acids such as malate and citrate [14], but others organic acids are secreted by plants upon the exposure of Al [15–17], and with chelating ability different to each organic acids with Al^3+^ (citrate> oxalate>malate) [18]. In this context, anion channels are responsible to confer tolerance to aluminum due to the efflux of Al^3+^ chelating malate/citrate anions via these channels, usually stimulated by Al^3+^ present in the rhizosphere. Two families of anion channels that confer Al tolerance are well described in the literature, the **Al**uminum-activated **M**alate **T**ransporter family (ALMT) and some members of the **M**ultidrug **a**nd **T**oxic Compound **E**xtrusion (MATE) family. ALMTs are implicated in the extrusion of Al via malate exudation, while MATE transporters are known to exudate citrate in the rhizosphere to chelate Al^3+^ [14, 19–21]. In sugarcane, none of these gene families members were identified to date. Since MATE transporters are members of a large gene family, in silico identification of *MATE* genes exclusively related to Al tolerance in sugarcane is very difficult, especially due to its high ploidy and genomic complexity [21]. Therefore, we aimed to identify and characterize homologous genes of *ALMT* in sugarcane (*Saccharum* spp.). Eleven *ALMT* genes were identified in the sugarcane genome, named *SoALMTs* (1 to 11), and phylogenetic analysis divided *SoALMTs* into 4 different clades. The expression level of *SoALMT* genes was also studied in roots of sugarcane plants in the presence or absence of Al. The results demonstrated that sugarcane plants overexpressing *SbMATE* exudated citrate to the rhizosphere and presented sustained root growth in the presence of Al, confirming the tolerance of the transgenic plants.

These results altogether represent a promising alternative for sugarcane expansion in Brazil and other areas with aluminum toxicity in poor and acid soils.

## Results

### Identification and characterization of *SoALMT*

**Fig. 1:**
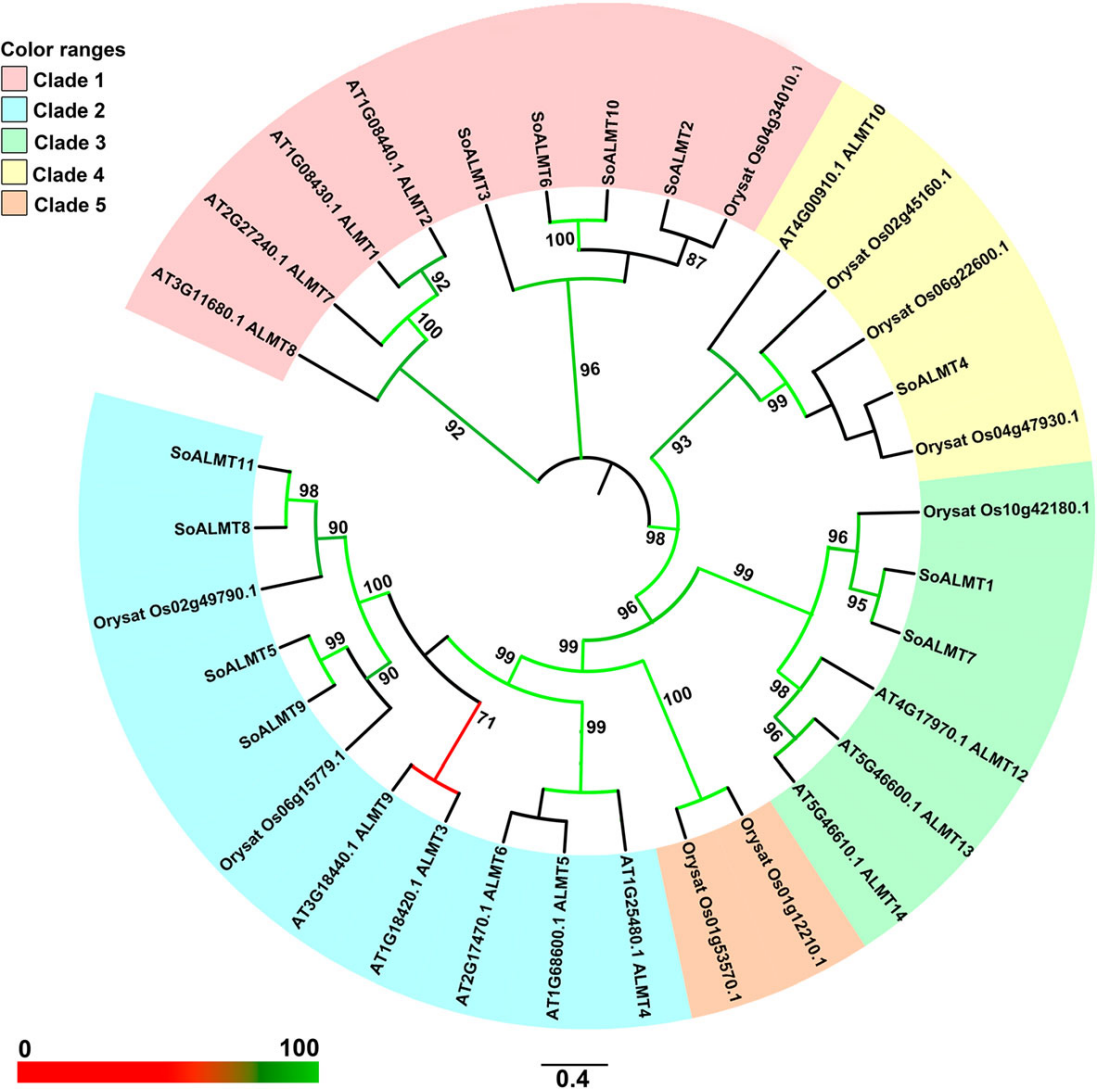
Maximum likelihood phylogeny of *ALMT*s in sugarcane and of plants. The bootstap values are represent in color branch scale and values > 70 are highlighted in the figure

**Fig. 2:**
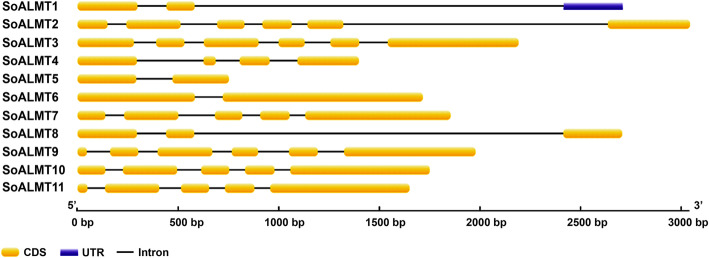
Intron/exon pattern of *SoALMT* gene. Exons and introns are shown as yellow boxes and thin lines, respectively

A total of 11 *ALMTs* with high similarity with previously identified *ALMTs* were found using the tool tblastn and the presence of Pfam PF11744 were identified. The putative *ALMTs* were numbered sequentially from 1 to 11, with prefix of the species *Saccharum officinarum* (*SoALMT*). The sequences data were deposited in the GenBank databases access MH137222 to MH137232. The size of *SoALMT* varied from 190 to 539 aa, with MW ranging from 6.04 to 59.11 KDa and pI from 5.36 to 50.77 (Table 1). All *SoALMT* contain at least one PF11744, with exception to *SoALMT2* and *SoALMT4* that presented two PF11744 (Table 1). Phylogenetic analysis classified the *SoALMT* into four different clades. *SoALMT2*, *SoALMT3*, *SoALMT6* and *SoALMT10* belong to clade 1; *So*ALMT5, *So*ALMT8, *So*ALMT9 and *So*ALMT11 were characterized as clade 2, while *SoALMT1* and *SoALMT7* as clade 3 and *SoALMT4* in the clade 4 (Fig. 1). *Saccharum officinarum ALMTs* did not show any representative member in clade 5. A variable number of introns were observed in *SoALMT*, varying from 1 to 5, with no correlation with phylogenetic classification (Table 1, Fig. 2). In general, three conserved motifs were observed in *SoALMTs*, with exception to the genes *SoALMT3*, which did not demonstrate conserved domains, and *SoALMT6*/*SoALMT10*, which belong to the clade 1 with the presence of only motif 1 (Additional file 1: Fig. S1). In the motif 1 it is possible to observe the conserved *ALMT* motif 2, characterized as *ALMT*/QUAC-like channel described by Motoda et al. (2007) [22] in *AtALMT*. With exception to *SoALMT4*, *SoALMT6* and *SoALMT10*, the other *SoALMT* members are potentially localized in the transmembrane (Table 1).

**Table 1:** Characterization of *Aluminum-activated Malate Transporter* (*ALMT*) genes from *Saccharum officinarum*

ID	Genome identification	aa	pI	MW (kDA)	PFAM	Putative localization	Phylogenetic classification	Introns number	GenBank ID
***SoALMT1***	evm.model.SCSP803280_000034311.1	539	9.01	59.11	38–499	TM	3	2	MH137222
***SoALMT2***	evm.model.SCSP803280_000052256.1	270	8.94	29.84	2–100; 97–219	TM	1	5	MH137223
***SoALMT3***	evm.model.SCSP803280_000052256.2	190	9.57	20.65	42–156	TM	1	5	MH137224
***SoALMT4***	evm.model.SCSP803280_000055753.1	525	5.36	56.31	60–123; 141–440	noTM	4	3	MH137225
***SoALMT5***	evm.model.SCSP803280_000074602.2	470	5.95	51.92	1–443	TM	2	1	MH137226
***SoALMT6***	evm.model.SCSP803280_000093844.2	242	5.39	26.31	43–242	noTM	1	1	MH137227
***SoALMT7***	evm.model.SCSP803280_000099098.2	462	8.46	51.04	1–422	TM	3	4	MH137228
***SoALMT8***	evm.model.SCSP803280_000107143.1	461	50.77	6.35	1–428	TM	2	2	MH137229
***SoALMT9***	evm.model.SCSP803280_000007339.1	429	46.57	8.91	1–301	TM	2	5	MH137230
***SoALMT10***	evm.model.SCSP803280_000007193.1	243	26.35	5.81	44–243	noTM	1	4	MH137231
***SoALMT11***	evm.model.SCSP803280_000146416.2	432	48.10	6.04	1–399	TM	2	4	MH137232

### Aluminum tolerance evaluation in transgenic plants

In this study, sugarcane RB855156 was transformed to overexpress the *Sorghum bicolor MATE* gene that encodes a citrate transporter. *SbMATE* is an Al^3+^ activated transport protein that confers Al tolerance to sorghum [20]. Seventeen different transgenic events were obtained and submitted to {0} and {505.9} μM Al^3+^ for over seven weeks. The pH of the treatments in the presence or absence of Al was measured daily and kept at 4.2 to guarantee the predominance of the free trivalent Al species. The Al concentration adopted in the present work was based on Oliveira (2012) [23] and other studies using hydroponic system to evaluate tolerance to Al in other crops [24–27]. After the initial screening, two sugarcane independent transgenic events with distinct responses to Al were chosen for further detailed studies (Events 1 and 2).

**Fig. 3:**
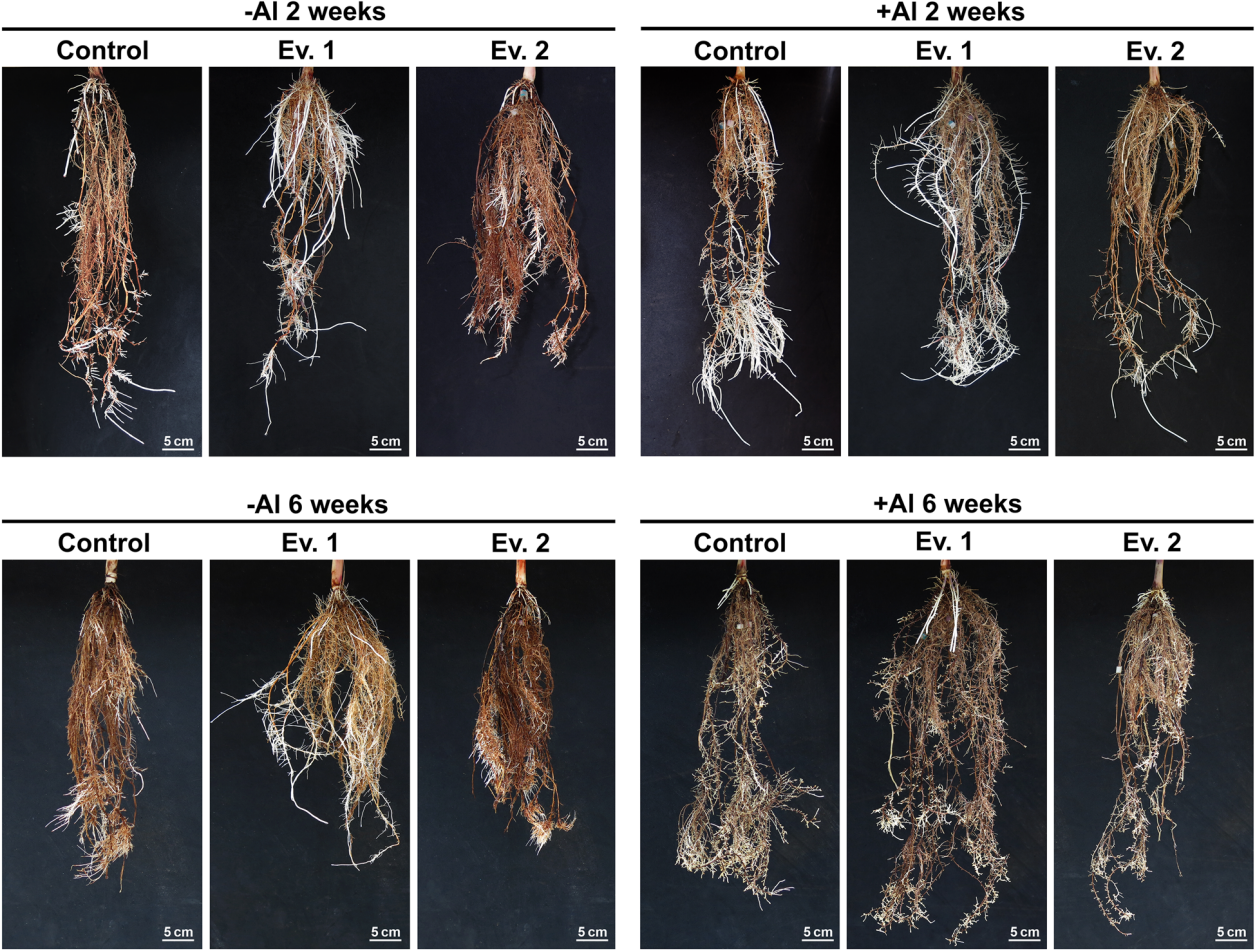
Sugarcane transgenic events overexpressing *SbMATE* and NT plants in the absence (−Al; left panel) and after 2 weeks and six weeks of exposure to {505.9} μM Al^3+^ (+Al; right panel)

**Fig. 4:**
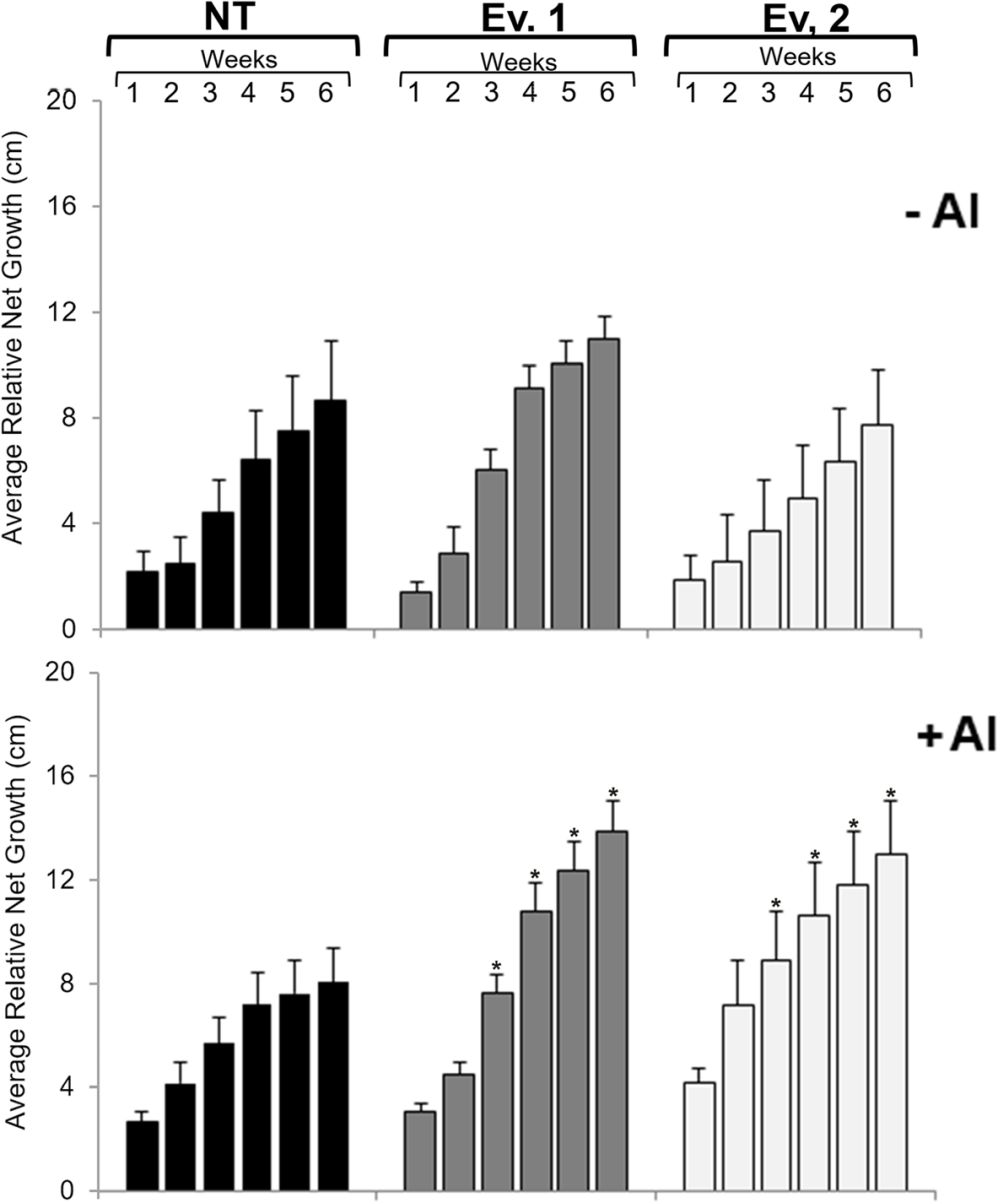
Relative Root Net Growth of sugarcane transgenic events *SbMATE* and NT plants grown under the absence (−Al) or presence (+Al) of {505.9} μM Al^3+^ over six weeks. The length of the roots was measured before and after each week of growth in the Hoagland’s half concentration solution with (+Al) and without (−Al) aluminum (*n* = 6 plantlets). *Significantly different at *p* < 0.05 between NT and transgenic plants

Figure 3 shows the phenotypic characteristics of roots under presence or absence of Al. Plants were photographed after 2 weeks and at the end of the experiment (after 6 weeks) in hydroponic system. The formation of brown spots in the roots was also observed, possibly due to oxidative stress or phenolic compounds accumulation [28]. Interestingly, roots from plants submitted to Al showed increased growth and only moderate symptoms of oxidative damage, even in NT plants, suggesting that cultivar RB855156 is tolerant to Al to some extent. However, sustained root growth was only achieved in transgenic plants overexpressing *SbMATE* (Fig. 4). Under Al treatment, relative net growth (RNG) of roots in transgenic plants increased by an average of 40% over the six weeks when compared to NT plants.

### Hematoxylin staining

**Fig. 5:**
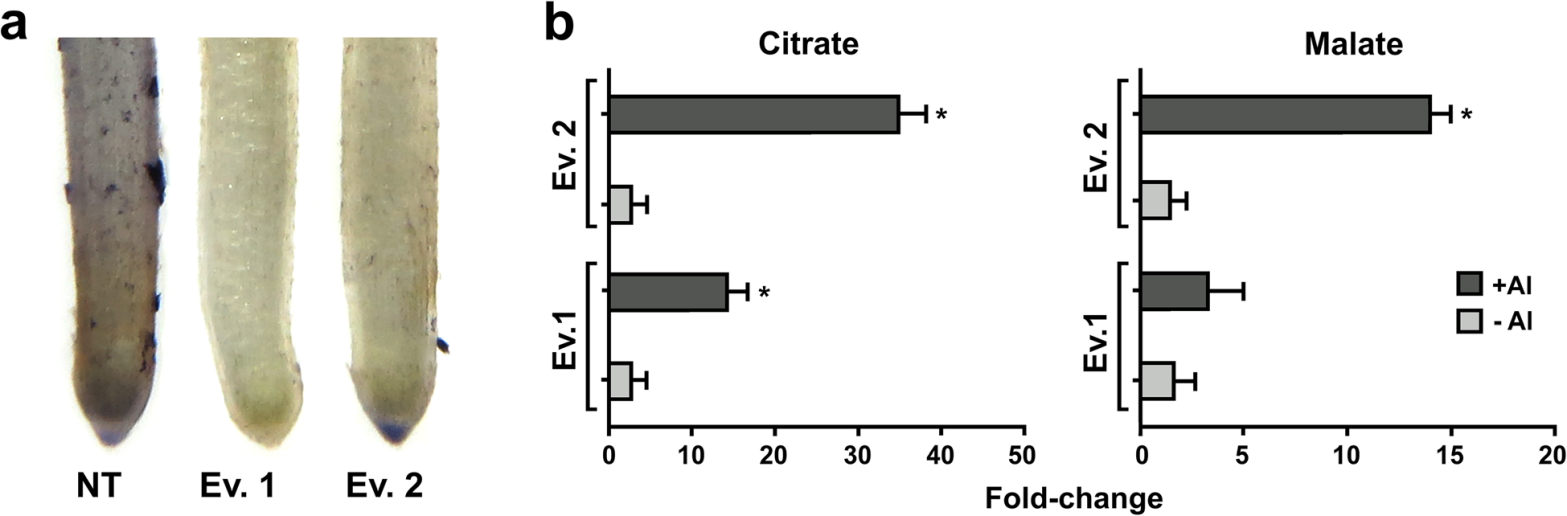
**(a)** Hematoxylin staining after 24 h exposure to {505.9} μM Al^3+^ in root tips of sugarcane transgenic events overexpressing *SbMATE* and NT plant. **(b)** Citrate and malate abundance on root exudates in the absence and after 12 days of exposure to {505.9} μM Al^3+^. The organic acids were determined by gas chromatography/mass spectrometry (GC/MS). The data are represented by fold-change of the organic acids in transgenic events compared to control plants. *Significantly different at *p* < 0.001 between NT and transgenic plants

The hematoxylin staining occurs by the complexation of the dye with Al. This assay is commonly used for rapid and qualitative screening of plants that may be tolerant to Al [3, 23, 25–27, 29, 30]. The root apex of plants demonstrating high accumulation of Al shows intense purple staining in the presence of hematoxylin. It is observed in Fig. 5a that the root apex of sugarcane transgenic plants showed less staining under Al treatment when compared to NT plants. These results indicate that sugarcane overexpressing *SbMATE* could be more tolerant to Al when compared to NT plants.

### Root organic acid efflux

The analysis of organic acids exudation from sugarcane roots demonstrated that the overexpression of *SbMATE* was able to increase the levels of citrate and malate in transgenic plants when compared to control. After 12 days in hydroponic solution with the presence of {505.9} μM Al^3+^, the transgenic lines presented a 14-fold and a 3-fold citrate and malate exudation, respectively, when compared to NT plants (Fig. 5b).

### *SoMATE* expression analysis

To verify if the endogenous sugarcane *MATE* gene was involved in Al-induced responses, expression analysis of the closest sugarcane homolog *MATE* gene (*SoMATE*) related to the sorghum *MATE* gene (*SbMATE*) was performed. The expression analysis revealed that, in the presence of Al, *SoMATE* increased its expression by ~ 20-fold in roots of NT plants in comparison with NT plants grown in the absence of aluminum (Additional file 2: Fig. S2), suggesting that *SoMATE* transporter is involved in Al responses in sugarcane. As expected, the exogenous *SbMATE* gene was not expressed in roots of NT plants, but it was highly expressed in transgenic sugarcane lines (Additional file 2: Fig. S2).

### *SoALMT*s expression analysis

**Fig. 6:**
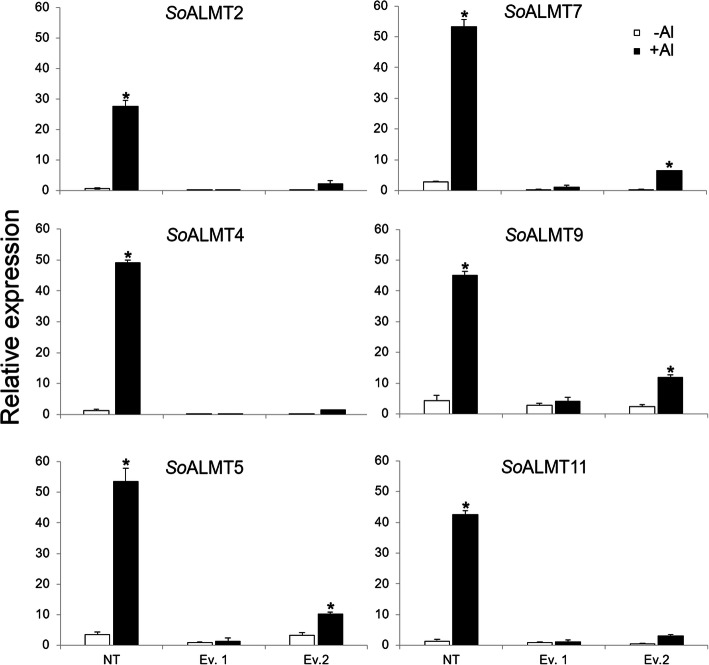
Relative gene expression of the *SoALMTs* in the NT and transgenic events submitted to {0} and {505.9} μM Al^3+^ after six weeks. *Significantly different at *p* < 0.05 between ­Al and + Al treatments in transgenic plants. Vertical bars show ± S.E. for *n* = 3

As described above, eleven genes corresponding to putative *ALMT* were identified in the sugarcane genome (Fig. 1). In NT plants, the expression of six of these genes drastically increased in the roots of sugarcane plants in the presence of Al (Fig. 6). *SoALMT*s *4*, *5*, *7* and *9* had their transcript levels increased by ~ 50-fold in roots of NT plants in the presence of Al, when compared to plants not exposed to the treatment, while *SoALMTs 2* and *11* increased their expression levels by ~ 30 and 40-fold in the same conditions, respectively (Fig. 6). The expression levels of *SoALMTs* in transgenic plants was not significantly increased in the presence of Al, suggesting that in plants overexpressing *SbMATE,* citrate is the predominant organic acid exudated.

### Expression analysis of aluminum-responsive genes

**Fig. 7:**
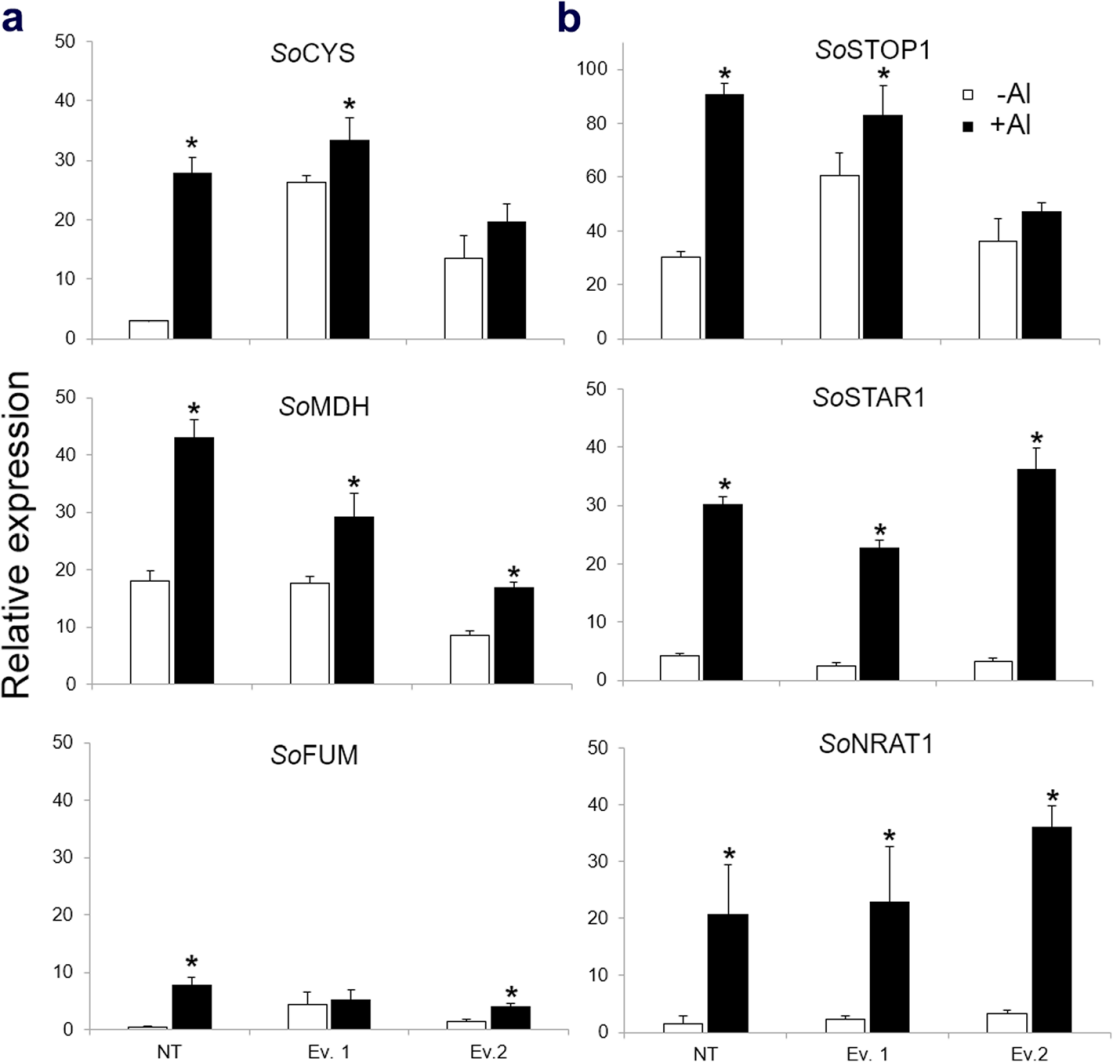
Relative gene expression levels of the *SoCSY* (CSY), *SoMDH* (MDH), *SoFUM (*FUM), *SoSTOP1, SoSTAR1* and *SoNRAT1* in the sugarcane transgenic events and NT plants submitted to {0} and {505.9} μM Al^3+^ after seven weeks. *Significantly different at *p* < 0.05 between ­Al and + Al treatments of the NT and transgenic plants. Vertical bars show ± S.E. for *n* = 3

Citrate and malate are the main organic acids secreted to the rhizosphere under Al stress and their production usually results in a substantial carbon cost for the plants [31]. Thus, the exudation process should be strictly regulated to maintain plant homeostasis. In this context, the expression levels of some genes involved in the tricarboxylic acid pathway such as citrate synthase (*SoCYS*), malate dehydrogenase (*SoMDH*) and fumarate dehydrogenase (*SoFUM*) was investigated to verify whether transgenic plants overexpressing *SbMATE* could lead to major impacts on primary metabolism. High expression levels of *SoMDH* could be observed even in the absence of Al both for NT and transgenic lines, indicating that basal transcript levels of MDH is high in sugarcane under our control conditions (Fig. 7a). However, the presence of Al drastically increased the transcript levels of *SoMDH*, which were ~ 20 and 10-fold higher in NT plants and in transgenic events, respectively, when compared to control conditions. These results corroborate the high expression levels verified in malate aluminum transporters (*SoALMTs*) in the presence of Al (Fig. 6). *SoCYS* transcript levels were relatively low in NT plants in the absence of Al but increased by ~ 30-fold in the presence of the metal (Fig. 7a). In contrast, transgenic plants produced high levels of *SoCYS* transcripts under normal conditions, as expected for plants constitutively overexpressing *SbMATE*, which exudate citrate constitutively. In the presence of Al, transgenic plants showed moderate increase in *SoCYS* expression levels (Fig. 7a). *SoFUM* transcription levels showed no significant change in either conditions tested, with exception to NT plants submitted to Al that presented transcription levels ~ 8-fold higher than NT plants under control conditions (Fig. 7).

*STOP1* is a zinc-finger transcription factor essential for Arabidopsis tolerance to Al stress [32]. The homologous gene of *STOP1* was identified in the sugarcane genome (*SoSTOP1*) and its transcription levels were studied in roots of NT and transgenic plants in the presence or absence of Al. The expression level of *SoSTOP1* increased by ~ 60-fold in plants under Al stress, especially in NT plants (Fig. 7b). *SoSTOP1* transcription levels in *SbMATE* plants were higher when compared to NT plants in the absence of Al and presented only moderate increase in transgenic plants submitted to Al (Fig. 7b).

*STAR1* is a bacterial-type ABC transporter involved in Al tolerance mechanism in rice [33]. The putative gene encoding *STAR1* was identified in the sugarcane genome (*SoSTAR1*) to verify its transcription levels in our experimental conditions. *SoSTAR1* expression is highly regulated by Al in both NT and transgenic plants, where the expression levels drastically increased (Fig. 7b).

The homologous gene encoding a plasma membrane Al^3+^ transporter (*NRAT1*), located in root apical cells and responsible for aluminum tolerance in rice [34, 35], was also identified in the sugarcane genome (*SoNRAT1*) and its expression levels was examined in roots of sugarcane plants grown in hydroponic conditions. *SoNRAT1* is highly responsive to Al, since roots of plants submitted to the metal presented higher levels of *SoNRAT1* transcripts compared to non-treated plants (Fig. 7b). Overall, these results suggest that some Al responsive genes are present in roots of sugarcane and these genes are highly responsive to the stress.

## Discussion

A biochemical mechanism used by plants to cope with Al stress involves the activation of membrane transporters responsible for organic acids secretion from the root apex to the rhizosphere. These organic acids form non-phytotoxic stable complexes with Al^3+^, preventing its absorption by the roots [4, 35–37]. The ALMTs proteins are among the transporters that play pivotal roles in the adaptation to acid soils [4]. These proteins exudate malate to the rhizosphere in the presence of Al, conferring tolerance, via chelation of Al^3+^ [4]. To the best of our knowledge, *ALMT* gene family members were not identified in sugarcane to date. Genome-wide analysis of the recently released sugarcane genome [38] identified 11 *ALMT* genes in sugarcane, which were phylogenetically divided into 4 different clades (Fig. 1). The study of the expression pattern of these different *ALMT* genes confirmed the involvement of the identified transporters in Al responses in sugarcane, since high levels of *SoALMT* transcripts was observed in roots of NT plants exposed to the metal (Fig. 6).

However, the expression of *SoALMTs 1*, *3*, *6*, *8* and *10* were not observed in roots of sugarcane in our experimental conditions, possibly because these genes are expressed in other tissues that were not studied in this work. ALMT proteins are also known to regulate several physiological responses in plants such as guard cell regulation, anion homeostasis, fruit quality, seed development and microbe signaling network [14]. Therefore, these transporters are present and expressed in different plant tissues and different developmental stages.

In sorghum, a membrane transporter gene belonging to the multidrug and toxic compound extrusion (*MATE*) family was identified and characterized as an Al-activated citrate transporter gene responsible for the Al-tolerance in this crop, and the overexpression of *SbMATE* conferred tolerance in Arabidopsis plants [20]. Moreover, the overexpression of close homolog of *SbMATE*, the *Brachypodim distachyon MATE* gene (*BdMATE*), in *Setaria viridis* conferred tolerance to Al. *S. viridis* is a C4 plant that is emerging as a model for grasses [3, 39]. Based on these studies, transgenic sugarcane lines constitutively overexpressing the *SbMATE* gene were generated to verify if the transgenic plants could demonstrate increased tolerance to Al. Sugarcane cv. RB855156 was successfully transformed to overexpress the *SbMATE* gene driven by *ZmUBi1* promoter using a protocol developed by our group [40]. Seventeen independent transgenic events were generated and screened for Al tolerance in plants growing in a hydroponic system (Additional file 3: Fig. S3), supplemented with an established concentration of Al^3+^ activity. Two out of the seventeen events demonstrated significant sustained root growth under Al treatment when compared to NT plants and used for further detailed analysis.

In hydroponic conditions, roots from both NT and transgenic plants grown in Hoagland’s solution in the absence of Al developed a brownish coloration after 2 weeks (Fig. 3), which appears to be correspondent to oxidative damage or accumulation of phenolic compounds [28]. Interestingly, in the presence of Al, sugarcane roots became vigorous with decreased symptoms of oxidative stress, indicating that the cultivar used for our studies is, at least to some extent, tolerant to Al. However, NT plants were unable to sustain root growth in the presence of Al over the period of the experiment, while the two transgenic events tested were able to maintain root growth (Fig. 4), in addition to increased number of adventitious roots (Fig. 3), indicating that *SbMATE* plants might be more tolerant to the metal when compared to NT plants. This phenomenon could be due to efflux of organic acids, in this case, citrate, and other mechanisms involved in tolerance aluminum investigated in this work, and as reported with other species as corn, sorghum, soybean, wheat, barley and Setaria [3, 17, 20, 41–43]. In addition, hematoxylin staining revealed that transgenic sugarcane roots did not accumulate Al in their apex, as indicated by lack of the purple coloration typical of the interaction between Al and the dye (Fig. 5a). It is known from previous studies that commonalities exist between Al and oxidative stress-induced gene expression in sugarcane apical roots, with several antioxidant genes upregulated under Al stress [44]. The high levels of antioxidant gene expression under Al treatment could explain the loss of oxidative damage symptoms observed in roots of sugarcane cv. RB855156 after Al treatment. Moreover, commercial cultivars of sugarcane are generally regarded as tolerant to Al, due to extensive breeding that has culminated with modern cultivars such as RB855156 [23, 45, 46]. As discussed by Guo et al. (2017) [47], which demonstrated root adaptive responses to different Al-treated *Citrus* cultivars, other factors could be responsible for Al-tolerance in plants, in addition to the antioxidant capacity. These factors include higher external Al detoxification capacity via enhanced Al-induced secretion of organic acid anions, a more efficient chelation system in roots, higher capacity to maintain the cellular phosphorus homeostasis by enhancing phosphorus acquisition and utilization, higher adaptive responses to Al concerning cell wall, cytoskeleton and carbohydrate metabolism and upregulation of genes related to fatty acid and aminoacid metabolism [37]. Despite that, sugarcane plants overexpressing *SbMATE*, when submitted to Al, are able to induce signaling pathways that promote root growth. Besides that, the action of the *SbMATE* transporter, as well as *ALMT1*, is specifically activated by the presence of Al ions, therefore, other benefits of OA secretion in roots, such as the improvement of P uptake, might be better observed in plant growing in presence of Al [34]. Another possible explanation is the fact that Al ions work as bacteriostatic and fungistatic, therefore, roots are less impacted by growth of opportunistic microorganisms in nutrient solution. Currently, we do not have any experimental evidence that this phenomenon is occurring, but in the next steps of Al response studies in sugarcane we will carefully address the mechanisms through which these plants are demonstrating improved Al tolerance. However, despite the high tolerance of sugarcane to Al, the extent and severity of soil acidification after intensive cultivation indicates that even slight susceptibility to the metal may result in severe economic losses [44–46]. Thus, the development of varieties with improved tolerance to Al is pivotal to ensure a suitable harvest.

Organic acids (OAs) secretion from the root apex to the rhizosphere is an important mechanism used by plants to cope with Al stress as OAs form non-phytotoxic stable complexes with Al^3+^, preventing its absorption by the roots [47]. However, OAs are also important components of plant primary metabolism. Malate, fumarate, lactate and citrate, produced via tricarboxylic acid pathway (TCA), are among organic acids of fundamental importance for several biochemical pathways, including energy production, formation of precursors for aminoacid biosynthesis and in modulating adaptation to the environment at the whole plant level [14, 48]. In this context, a balance between the positive effects of OA release and the disadvantage of losing valuable carbon sources is a desirable feature in the selection of transgenic plants constitutively expressing transporters involved in exudation of OAs. Thus, the transcription levels of several Al-responsive genes in roots of sugarcane in the presence or absence of the metal, including genes encoding intermediate enzymes of the TCA pathway, such as citrate synthase (CYS), malate dehydrogenase (MDH) and fumarate dehydrogenase (FUM) was investigated. First, these genes were identified in the sugarcane genome to perform qRT-PCR analysis to determine their expression levels in hydroponically grown NT or transgenic plants, submitted or not to Al stress (Fig. 7a). As expected, *SbMATE* plants showed higher levels of *SoCYS* expression even in the absence of Al, possibly due to the increased concentration of citrate exudation.

In the presence of Al, roots of both NT and transgenic plants increased their *SoCYS* transcription levels, indicating that citrate production is involved in Al stress responses in sugarcane. Malate dehydrogenase (*SoMDH*) gene expression levels were drastically increased in roots of NT and transgenic plants submitted to Al when compared with hydroponically grown plants in the absence of the metal, suggesting the involvement of malate in sugarcane responses to Al. These results corroborate with the high transcription levels of *SoALMTs* verified in roots of sugarcane submitted to Al (Fig. 6). Interestingly, higher malate exudation was found in transgenic plants in comparison to control in the presence of Al (Fig. 5b). It was found that citrate exudation is accompanied by malate efflux in transgenic events, possibly indicating a biochemical compensatory mechanism of organic acids in transgenic plants, as the efflux of these compounds can promote pH imbalance in the cell. Fumarate dehydrogenase (*SoFUM*) gene expression levels increased in roots of NT plants submitted to Al, but it was not significantly altered in roots of transgenic plants under the stress. These data demonstrate that sugarcane overexpressing *SbMATE* might be using TCA pathway intermediates in a greater extent compared to NT plants. Indeed, increased Al resistance correlates with higher rates of citrate and malate exudation in several plant species, as observed for snapbean, maize and *Cassia tora* [49–52] and it appears to be the case also in sugarcane (Fig. 5b). Moreover, alternative glycolytic pathway genes were also differentially expressed in Al-treated roots of two *Citrus* cultivars, which demonstrate differential responses to Al and phosphorous [53]. These results reinforce that glycolytic pathways are actively involved in Al responses in different plant species. It is worth mentioning that the increase of organic acid secretion is not always the main mechanism for Al-tolerance in plants, but contributes to reduce Al^3+^ in the cytosol, which is an important internal mechanism to improve the tolerance of plants to Al. However, the precise mechanism of Al tolerance and the molecular mechanisms of Al tolerance in plants are still not clear, due to Al interference in multiple sites in the apoplast and symplast, and the complexity of plant self-defense.

The mechanisms of Al toxicity and crop tolerance to Al mainly depends on the study of roots. For instance, phosphorus (P) supply can alleviate Al-toxicity through increasing immobilization of Al in roots and P levels in seedlings rather than through increasing of OA anion secretion in *Citrus* [54]. In this regard, it is important to verify soil conditions to improve Al-tolerance in different plant species.

Finally, the expression pattern of some genes known to be associated with Al responses was also investigated. The orthologous genes for *STOP1*, *STAR1* and *NRAT1* were identified in the sugarcane genome and their transcription levels were investigated as described above for the TCA pathway genes. *STOP1* is a zinc-finger transcription factor that co-regulates a key gene in Al tolerance mechanism in Arabidopsis and appeared to be required for *AtMATE* expression and Al-activated citrate exudation [32]. It is known that the transcription regulation exerted by *STOP1* can be activated not only by Al, but also by low pH [55]. In rice studies, Arenhart et al. (2014) [33] found that *STAR1* gene was the only ABC gene whose transcription level was increased in the Al-treated NT plants, but decreased in the *ASR5*_RNAi transgenic plants compared to the untreated NT plants. *STAR1* was one of the *ASR5* target genes identified in the ChIP-Seq analysis, and *ASR5* binding to the *STAR1* promoter region was confirmed via in vitro DNA-binding assays [33]. This disruption resulted in hypersensitivity to Al toxicity [56]. NRAT1 is a plasma membrane Al^3+^ transporter located in root apical cells and responsible for Al tolerance in rice. As demonstrated by Xia et al. (2010) [34], knockout of *NRAT1* resulted in decreased Al uptake, increased Al binding to cell wall, and enhanced Al sensitivity. The expression of *NRAT1* is up-regulated by Al in the roots and regulated by a C_2_H_2_ zinc finger transcription factor (*ART1*) in rice, and this mechanism is required for a prior step of final Al detoxification through sequestration of Al into vacuoles [44]. The transcription levels of *SoSTOP1*, *SoSTAR1* and *SoNRAT1* were drastically increased in roots of sugarcane submitted to Al treatment, suggesting their involvement in Al tolerance pathways in sugarcane.

Unfortunately, hydroponically grown sugarcane does not achieve developmental stages where important measurements such as sucrose content or biomass can be performed. These measurements are of fundamental importance to verify if *SbMATE* plants are suitable for agricultural purposes. Field trials performed in the *Cerrado* region of Brazil, using these candidate elite events, are currently underway to address these questions. It is also worth noticing that the detailed mechanism of sugarcane Al-tolerance was not the scope of the present study. The identification of genes possibly involved in Al responses in sugarcane such as So*ALMT* or TCA cycle genes might help to elucidate the mechanism of Al-tolerance in sugarcane.

## Conclusions

In conclusion, sugarcane plants constitutively overexpressing the *Sorghum bicolor MATE* gene (*SbMATE*) demonstrated improved tolerance to Al when compared to NT plants, characterized by sustained root growth and possible exclusion of Al from the root apex. In addition, in silico analysis and molecular studies identified potential new targets for Al tolerance in sugarcane. These results represent a promising alternative for agricultural expansion in Brazil and other areas with Al toxicity and acid soils.

## Methods

### SbMATE cloning

The sequence of the *Sorghum bicolor MATE* (Sb03g043890) gene was optimized using the preferred *Zea mays* codons to facilitate the synthesis of the gene in the cloning step, since the native *SbMATE* gene has a high GC content. In addition, a Kozak sequence (CCGAA-ATG) was added upstream of the coding region. Subsequently, alignments of the *SbMATE* and *oSbMATE* (codon optimized) sequences were performed using the Geneious software v. 2020.2 [57]. Alignments of nucleotide and aminoacid sequences were performed to demonstrate that the optimization step did not modify the final protein sequence, as shown in Additional file 4 Fig. S4 and Additional file 5: Fig. S5. The optimized sequence was synthesized and cloned into a binary vector by the company DNA Cloning Service (Germany).

### Identification of *ALMT* genes in sugarcane

To identify members of *Aluminum-activated Malate Transporters* (*ALMT*) genes in sugarcane, the keyword “ALMT” search was used to screen plant proteins in the National Center for Biotechnology Information (NCBI) bank. The protein sequences found were then used as query against the genome sequence of sugarcane (SP80–3280 cultivar) downloaded from GenBank accession number GC_002018215.1, using blastp with *e-value* cutoff set to 1e-10 to identify potential ALMTs. Redundant protein sequences were removed using a custom Perl program and remnant sequences were investigated for the presence of conserved domain PF11744 in the Pfam server v. 33 (http://pfam.xfam.org/). Proteins with the absence of PF11744 domain were removed of the dataset. The putative ALMTs had the characterization of molecular weight (MW), theoretical isoeletric point (pI) and protein length (aa) predicted using the tool Protparam (http://web.expasy.org/protparam), and position of Pfam domain using HMMSCAN v. 2.41.1 (https://www.ebi.ac.uk/Tools/hmmer/search/hmmscan).

Subcellular localization of sugarcane *ALMTs* (*SoALMT*) were determined using ProtComp 9.0 program (http://www.softberry.com/berry.phtml?topic=protcomppl&group=programs&subgroup=proloc). The exon/intron pattern and conserved evolutionary domains of putative *SoALMTs* were analyzed using the online softwares Gene Structure Display Server v. 2.0 (GSDS: http://gsds.cbi.pku.edu.ch), Multiple Em for Motif Elicitation (MEME v. 4.11.1) server software [58], FFPred v. 2.0 and MEMSAT v. 2.0 [59, 60].

### Phylogenetic analysis of ALMTs

To study the evolutionary relationship and classification of *SoALMT* and reference sequences previously segregated in five established clades (Orysat Os02g49790.1, Orysat Os06g15779.1, AT3G18440.1_ALMT9, AT1G18420.1_ALMT3, AT2G17470.1_ALMT6, AT1G68600.1_ALMT5, AT1G25480.1_ALMT4, Orysat Os01g53570.1, Orysat Os01g12210.1, AT5G46610.1_ALMT14, AT5G46600.1_ALMT13, AT4G17970.1_ALMT12, Orysat Os10g42180.1, Orysat Os04g47930.1, Orysat Os06g22600.1, Orysat Os02g45160.1, AT4G00910.1_ALMT10, Orysat Os04g34010.1, AT1G08440.1_ALMT2, AT1G08430.1_ALMT1, AT2G27240.1_ALMT7 and AT3G11680.1_ALMT8), the sequences were aligned using the Muscle and inferred in the FastTree v. 2.1.5 program [61]. The reference sequences were obtained from Dreyer et al. (2012) [62] and used as pattern to classification of *So*ALMT. The phylogenetic tree was visualized using the online software iTOL v.5 (https://itol.embl.de/).

### Genetic transformation of sugarcane

In this study we used the sugarcane cultivar RB855156, this sugarcane cultivar is in public domain and registered/provided by Ridesa (Rede interuniversitária para o desenvolvimento do setor sucroenergético) (https://www.ridesa.com.br/) and no deposited in none publicly available herbarium.

Immature top stalks of 6–9-month-old plants from sugarcane cultivar RB855156, were collected and introduced in vitro for induction and obtainment of the embryogenic callus, according to Basso et al. (2017) [40]. Embryogenic calli were selected and cultured at 3-week intervals on the same medium prior to bombardment [63, 64]. The embryogenic calli were selected and used for genetic transformation by biobalistics and the expression vector p7U was used for sugarcane transformation (DNA Cloning Service, Germany). This vector contains the *Sorghum bicolor MATE* gene with optimized codon (*oSbMATE*) under the control of *ZmUbi1* promoter. The selective marker is *bar* (phosphinothricin acetyl transferase) gene driven by *ZmUbi1* promoter, which confers resistance to glufosinate-ammonium herbicide (Additional file 3: Fig. S3a). The microprojectile suspension was prepared as described previously [65].

Following bombardment, calli were transferred to solid MS medium [66], supplemented with 20 g/L sucrose, 3 mg/L 2,4-dichlorophenoxyacetic acid (2,4-D), solidified with 4 g/L Phytagel™, supplemented with 250 mg/L cefatoxime sodium and incubated for 7 days in the dark at 27 ± 2 °C. Subsequently, calli were transferred to plates containing MSC3 medium [MSC3 consisting of MS salts and supplemented with 0.5 mg/L nicotinic acid, 0.5 mg/L pyridoxine HCl, 0.1 mg/L thiamine HCl, 2 mg/L glycine, 50 mg/L arginine, 0.15 mg/L citric acid, 27.25 g/L sucrose, 100 mg/L myo-inositol, 50 mg/L L-cysteine, 500 mg/L hydrolyzed casein, 3 mg/L 2,4-D, 0.6 mg/L copper sulfate, 50 mL/L commercial coconut water, 4 g/L Phytagel™, pH 5.8] plus 250 mg/L cefatoxime sodium and 3 mg/L of glufosinate-ammonium (LibertyLink™ herbicide, Bayer) as selective agent and the selected putative transgenic calli were used for plant regeneration, according to Basso et al. (2017) [40].

The regenerated plants were transferred to MS medium supplemented with 250 mg/L citric acid, 250 mg/L cefatoxime sodium, 3 mg/L glufosinate-ammonium, maintained in a growth chamber Conviron® Adaptis 1000TC (Conviron, Canada) in 16/8-h light/dark photoperiod at 100 μmol m^− 2^ s^− 1^and 27 ± 1 °C. The regenerated plants were acclimated in pots containing soil, commercial substrate Plantmax™ and vermiculite mixture (3:1:0.5) for 8 to 12 days in the growth chamber at 26 ± 2 °C, under a 16/8-h light/dark photoperiod at 400 μmol m^− 2^ s^− 1^ and 65% relative humidity [67]. Plants were transferred to a greenhouse and multiplied for subsequent Al tolerance assays. Non-transformed (NT) plants were used as control for all experiments described.

### Molecular analysis of transgenic events

Genomic DNA from regenerated plantlets resistant to glufosinate-ammonium was extracted using a modified CTAB method [67]. The gene insertion was confirmed by PCR using specific primers designed for *oSbMATE* amplification (Additional file 3: Fig. S3b).

Total RNA from roots apex was extracted using a LiCl method [68]. Samples were treated with RQ1 RNase-free DNAse according to the manufacturer’s instructions (Promega, Madison, WI, USA) and total RNA was quantified using a NanoDrop ND-1000 Spectrophotometer (Uniscience). RNA integrity was verified by agarose gel electrophoresis. The synthesis of the first strand cDNA was accomplished using the extracted RNA as template and the RevertAid™ First Strand cDNA Synthesis kit (Thermo Fisher Scientific). All steps were performed according to the manufacturer’s instructions. The qPCR analysis was carried out using Platinum® SYBR® Green PCR SuperMix-UDG with ROX (Invitrogen, Carlsberg, CA, USA) with synthesized single-stranded cDNA as template, using the protocol recommended by the StepOnePlusReal-Time PCR Systems (Applied Biosystems). The primers were designed using the software Primerquest (https://www.idtdna.com/primerquest/Home/Index), and their specificity was verified using electronic PCR (https://ncbiinsights.ncbi.nlm.nih.gov/2017/06/28/e-pcr-is-retiring-use-primer-blast/). The primers were synthesized and purchased from Integrated DNA technologies (IDT^®^), and their sequences are described in Additional file 6: Table S1.

Relative gene expression levels were calculated using the q-Gene [69] and the expression level was normalized using the reference genes *SoGAPDH* and *SoEF1,* according to Santiago et al. (2018) [70]. The geometrical mean of the relative quantities (RQs) was calculated using BestKeeper software v.1 [71]. Individual amplification efficiencies were established with LinRegPCR v. 11.0 using a window-of-linearity [72]. The experiment was performed using three biological replicates.

### Aluminum treatment assay and root growth measurement

Pre-budded seedlings of sugarcane (PBS) of seventeen transgenic events and NT plants were established from individual buds in tubes for plantlets containing vermiculite. After 60 days, the plants were selected according to their vigor and healthy root development and transferred to hydroponic system containing ½ Hoagland [73] nutrient solution. The plants were then acclimated for 7 days (Additional file 7: Fig. S6). Afterwards, the plants were submitted to Al treatment with 2.10 mM AlCl_3_, which corresponds to {505.9} μM of Al^3+^ free activity in hydroponic solution. The pH was adjusted to 4.2 and it was measured daily using a portable pH meter (Hanna HI9023 pH meter; Hanna Instruments Brazil Exp. E Imp. LTDA). Al^3+^ activity was estimated using the software GeoChem-EZ v. 1.0 [74]. The evaluation of root growth was performed every 7 days for 7 weeks.

Transgenic and NT plants were evaluated for liquid root growth in treatments in the presence or absence of Al as described in Ryan et al. (2009) [75]. In order to measure the relative net growth (RNG), the length of the roots was measured with a ruler before and after each week of growth in the nutrient solution with (+Al) and without (−Al) Al over seven weeks.

### Hematoxylin staining

The hematoxylin method was used to evaluate Al accumulation in sugarcane plants under Al treatment, which was performed as described above for root growth measurement. The protocol was based on Tang et al. (2000) [30]. Briefly, root tips of 3 cm in length from six plants of each transgenic event and NT plants grown in nutrient solution with the presence or absence of Al for 24 h were excised from the plantlets and gently shaken in 2 mL of distilled water for 60 min. The water was replaced by 2 mL of aqueous hematoxylin solution (0.2% hematoxylin and 0.02% potassium iodide, w/v) and samples were gently shaken for 15 min. Finally, the solution was replaced one more time by 2 mL distilled water, thereby repeating the first step. After staining, the roots were photographed under the stereomicroscope Leica Model S8APO.

### Measurements of citrate and malate efflux

Citrate/Malate efflux in the root apices of transgenic and NT plants grown hydroponically, as described above, were collected 12 days after the exposure to a nutrient solution containing 0 and 505.9 μM of Al^3+^. After the exposure period, the roots were washed with distilled water and the liquid collected was lyophilized for organic acids analysis. Samples were derivatized with 200 μl of pyridine and 50 μl of N-O-bis (trimethylsilyl) trifluoracetamide (BSTFA) during 1 h at 75 °C under agitation. The samples were centrifuged at 13000 rpm for 5 min and 100 μl were collected into sealed glass vials for analysis. The metabolic profile was performed injecting 1 μl into a gas chromatography–mass spectrometry (GC-MS) system (Agilent GC 6890 and MSD 5973 N series, Agilent, The United States), according to the method described by Centeno et al. (2016) [76] on a 30 m HP5 column with 0.25 mm of diameter and 0.25 μM film thickness (Supelco). Helium was used as carrier gas at a flow rate of 1 mL. min^− 1^. The analysis was performed under the following temperature program: 5 min of isothermal heating at 70 °C, followed by a 5 °C min^− 1^ oven temperature ramp to 310 °C, and a final 1 min of heating at 310 °C. Mass spectra were recorded at 2 scan s^− 1^ with a scanning range of 50–600 m/z. The peaks were identified using the NIST Mass Spectral Library. The data was expressed in fold-change between the transgenic lines and the NT in each condition.

### Statistical analysis

Experimental data were analyzed using randomized block design (RBD) with six replicates for each treatment ({0} and {505.9} μM Al^3+^). Samples for all analyses were collected after 7 weeks of treatment with exception to organic acid analysis where samples were collect after12 days of treatment. Differences among treatments per sample were analyzed using *t* test, considering *p* < 0.05 as significant.

## Supplementary Information

**Additional file 1 Supplementary Fig. 1** Conserved motifs of *So*ALMT proteins according to MEME software.

**Additional file 2 Supplementary Fig. 2** Relative gene expression of the *So*MATE (left panel) and *Sb*MATE (right panel) genes in the NT and transgenic events submitted to {0} and {505.9} μM Al^3+^ during six weeks. *Significantly different at *p* < 0.05 between - Al and + Al treatments of the NT and transgenic plants.

**Additional file 3 Supplementary Fig. 3 (a)** Schematic representation of the binary vector p7U (DNA Cloning Service, Germany). The vector contains the *Sorghum bicolor* MATE gene with optimized codon (*oSb*MATE) under the control of *Zm*Ubi1 promoter. The selective marker is *bar* (phosphinothricin acetyl transferase) gene under the control of the *Zm*Ubi1 promoter, confers resistance to glufosinate-ammonium herbicide. **(b)** Agarose gel electrophoresis showing the expected amplicon of *Sb*MATE gene (301 bp) in the transgenic events.

**Additional file 4 Supplementary Fig. 4.** Alignment of the nucleotides sequences. Nucleotides alignment of optimized (*oSb*MATE) and original *Sorghum bicolor* MATE **(***Sb*MATE **-** SbMATE03g043890**)** sequences generated by Geneious software (Kearse et al., 2012). (*) Symbols under the alignments indicate identical.

**Additional file 5 Supplementary Fig. 5.** Alignment of the deduced amino acid sequences. Geneious software (Kearse et al., 2012**)** alignment of the deduced amino acid sequences of optimized (o*Sb*MATE) and original *Sorghum bicolor* MATE **(***Sb*MATE **-** SbMATE03g043890**)** sequences. (*) Symbols under the alignments indicate identical.

**Additional file 6 Supplementary Table 1** Sequence of the pair of primers used for PCR and qPCR analysis.

**Additional file 7 Supplementary Fig. 6 (a)** Hydroponics system of sugarcane in greenhouse. **(b)** Sugarcane transgenic plants *Sb*MATE and NT plants in the hydroponic system, before aluminum treatment.

## Data Availability

The *ALMTs*-sequencing data reported in this paper have been deposited in National Genomics Data Center, in the GenBank databases under accession number(s) MH136222 to MH137232. The remaining datasets used to support the conclusions of this article are included within the article and its additional files.

## Abbreviations

Al: Aluminum
pH: potential of hydrogen
ALMT: Aluminum-activated Malate Transporter
MATE: Multidrug and Toxic Compound Extrusion
NT: Non-transformed
OA: Organic acid
TCA: Tricarboxylic acid
CYS: Citrate synthase
MDH: Malate dehydrogenase
FUM: Fumarate dehydrogenase
qRT-PCR: Quantitative real-time polymerase chain reaction
NCBI: National Center for Biotechnology Information
MW: Molecular weight
ZmUbi1: Polyubiquitin promoter Ubi-1
2,4-D: 2,4-dichlorophenoxyacetic acid
CTAB: Cetyl trimethylammonium bromide
PBS: Pre-budded seedlings
BSTFA: N-O-bis (trimethylsilyl) trifluoracetamide
GC-MS: Gas chromatography–mass spectrometry
RBD: Randomized block design
