# Supplementary figures and images for: Enhanced aluminum tolerance in sugarcane: evaluation of SbMATE overexpression and genome-wide identification of ALMTs in Saccharum spp
Source: BMC Plant Biol. 2021 Jun 29;21:300. doi: 10.1186/s12870-021-02975-x (PMC8240408; doi:10.1186/s12870-021-02975-x)

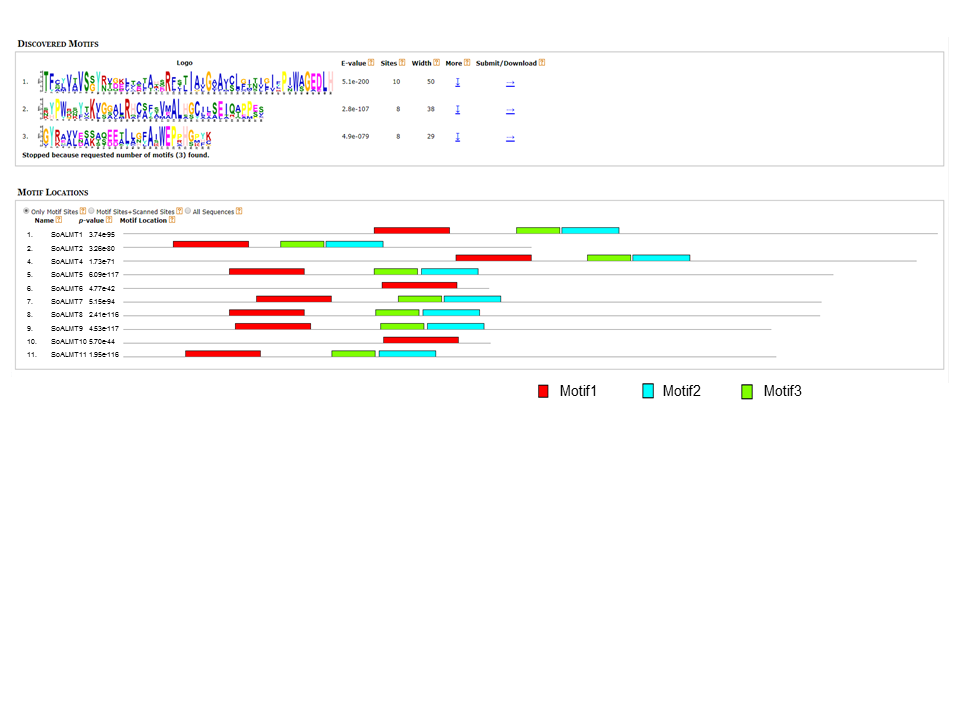


**Supplementary Fig. 1** Conserved motifs of *So*ALMT proteins according to MEME software.

Supplement: Supplementary file 1 — Additional file 1 Supplementary Fig. 1 Conserved motifs of SoALMT proteins according to MEME software. [file 12870_2021_2975_MOESM1_ESM.doc]
