# Supplementary material for: Enhanced aluminum tolerance in sugarcane: evaluation of SbMATE overexpression and genome-wide identification of ALMTs in Saccharum spp
Source: BMC Plant Biol. 2021 Jun 29;21:300. doi: 10.1186/s12870-021-02975-x (PMC8240408; doi:10.1186/s12870-021-02975-x)
Supplement: Supplementary file 2 — Additional file 2 Supplementary Fig. 2 Relative gene expression of the SoMATE (left panel) and SbMATE (right panel) genes in the NT and transgenic events submitted to {0} and {505.9} μM Al3+ during six weeks. *Significantly different at p < 0.05 between - Al and + Al treatments of the NT and transgenic plants. [file 12870_2021_2975_MOESM2_ESM.doc]

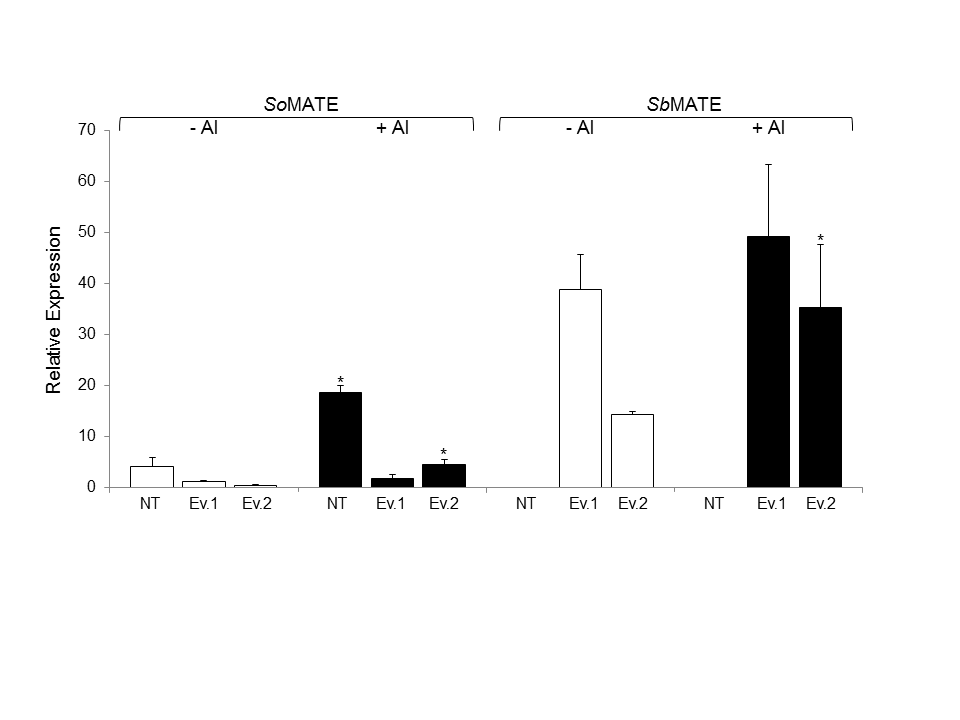


**Supplementary Fig. 2** Relative gene expression of the *So*MATE (left panel) and *Sb*MATE (right panel) genes in the NT and transgenic events submitted to {0} and {505.9} μM Al^3+^ during six weeks. *Significantly different at *p* < 0.05 between - ­Al and +Al treatments of the NT and transgenic plants.
