# Supplementary material for: Enhanced aluminum tolerance in sugarcane: evaluation of SbMATE overexpression and genome-wide identification of ALMTs in Saccharum spp
Source: BMC Plant Biol. 2021 Jun 29;21:300. doi: 10.1186/s12870-021-02975-x (PMC8240408; doi:10.1186/s12870-021-02975-x)
Supplement: Supplementary file 3 — Additional file 3 Supplementary Fig. 3 (a) Schematic representation of the binary vector p7U (DNA Cloning Service, Germany). The vector contains the Sorghum bicolor MATE gene with optimized codon (oSbMATE) under the control of ZmUbi1 promoter. The selective marker is bar (phosphinothricin acetyl transferase) gene under the control of the ZmUbi1 promoter, confers resistance to glufosinate-ammonium herbicide. (b) Agarose gel electrophoresis showing the expected amplicon of SbMATE gene (301 bp) in the transgenic events. [file 12870_2021_2975_MOESM3_ESM.doc]

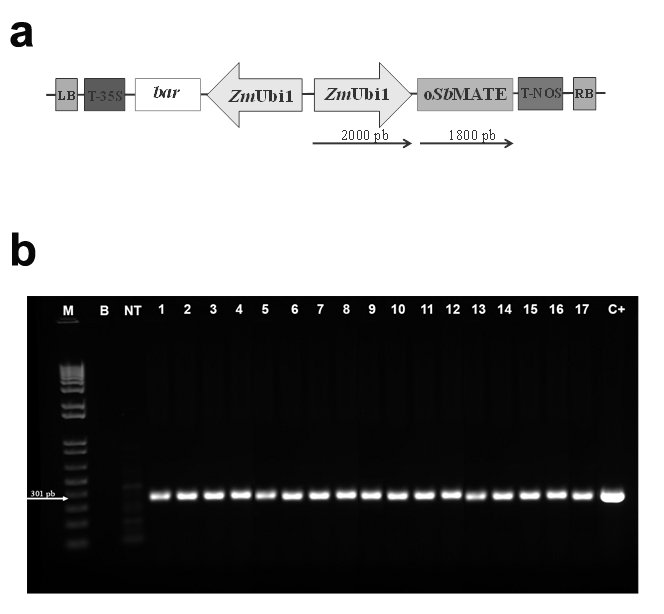


**Supplementary Fig. 3 (a)**Schematic representation of the binary vector p7U (DNA Cloning Service, Germany). The vector contains the *Sorghum bicolor* MATE gene with optimized codon (*oSb*MATE) under the control of *Zm*Ubi1 promoter. The selective marker is *bar* (phosphinothricin acetyl transferase) gene under the control of the *Zm*Ubi1 promoter, confers resistance to glufosinate-ammonium herbicide. **(b)** Agarose gel electrophoresis showing the expected amplicon of *Sb*MATE gene (301 bp) in the transgenic events.
