# Supplementary material for: Enhanced aluminum tolerance in sugarcane: evaluation of SbMATE overexpression and genome-wide identification of ALMTs in Saccharum spp
Source: BMC Plant Biol. 2021 Jun 29;21:300. doi: 10.1186/s12870-021-02975-x (PMC8240408; doi:10.1186/s12870-021-02975-x)
Supplement: Supplementary file 4 — Additional file 4 Supplementary Fig. 4. Alignment of the nucleotides sequences. Nucleotides alignment of optimized (oSbMATE) and original Sorghum bicolor MATE (SbMATE - SbMATE03g043890) sequences generated by Geneious software (Kearse et al., 2012). (*) Symbols under the alignments indicate identical. [file 12870_2021_2975_MOESM4_ESM.doc]

SbMATE ATGGAGGAACACCGGTCACCAGCTCACGCCAAGCCCGAGGCCGAGCAGCCACCGCAGCAG

oSbMATE atggaggaacacaggagcccggcccatgcgaagcctgaggctgagcagccaccccaacag

************ ** ** ** ** ** ***** ***** *********** ** ***

SbMATE CAGGTGCCGGCGGCGATGGCGGTGGCAGTGGCAGTGGACGTCGCTGCTCCAGCAGCGCTA

oSbMATE caagtgccggcagccatggcggtcgctgttgccgtggacgtcgcagcgcctgccgctctg

** ******** ** ******** ** ** ** *********** ** ** ** ** **

SbMATE CAGAATAGTACTGCGGCTCCTGCTGAGAACGGGGACGTCGCTGCTGCGGGCGCGGCAGAG

oSbMATE cagaactccaccgccgcgcccgcagaaaatggcgatgttgctgccgccggggcggcagag

***** ** ** ** ** ** ** ** ** ** ** ***** ** ** *********

SbMATE AATGGTACTGCGGCTTCCGCTGCGAACGGGGACGGCGGCGGCTCGGAGCTGCTCGGCGGT

oSbMATE aacggtactgctgcgtctgccgctaatggcgacggaggcgggtcagagctccttggtggc

** ******** ** ** ** ** ** ** ***** ***** ** ***** ** ** **

SbMATE CCACGCTGGACGGGGCTGCACCTTTTCGTGATGAACATCCGGAGCGTGTTCAAGCTGGAC

oSbMATE ccacgctggacaggattgcacctgttcgtgatgaacatccggtcggtctttaaactcgat

*********** ** ******* ****************** ** ** ** ** **

SbMATE GAGCTCGGCGCGGAGGTGCTGGGCATCGCGGTGCCGGCGTCGCTGGCGCTGACGGCCGAC

oSbMATE gaacttggcgccgaagtgctggggattgcagttccggcgagtctcgccttgacggctgac

** ** ***** ** ******** ** ** ** ****** ** ** ******* ***

SbMATE CCGCTCGCCTCGCTGATCGACACGGCCTTCATCGGCCGGCTGGGGTCCGTGGAGATCGCG

oSbMATE cctctggcgtccctcatcgataccgcattcatcggcagacttggtagcgtcgagattgcc

** ** ** ** ** ***** ** ** ********* * ** ** *** ***** **

SbMATE GCCGTGGGCGTCGCCATCGCGGTGTTCAACCAGGTCATGAAGGTGTGCATCTACCCGCTC

oSbMATE gctgtgggagtcgcgatcgccgtgttcaaccaagttatgaaagtgtgcatttaccccttg

** ***** ***** ***** *********** ** ***** ******** ***** *

SbMATE GTCAGCGTCACCACGTCGTTCGTCGCGGAGGAGGACGCCGTGCTCAGCAAAGGCGGCGCC

oSbMATE gtctctgttactacatcatttgtggcagaggaggacgccgtcctctcgaagggcggggcg

*** ** ** ** ** ** ** ** ************** *** ** ***** **

SbMATE AAGGTCATCGACAACGGAGAAGAAGAAGAAGAATTAGAAGCGGGACAAGTTGGCCCGGAG

oSbMATE aaagtgatcgacaatggcgaggaagaggaggaactggaggctggtcaagtcggaccagag

** ** ******** ** ** ***** ** *** * ** ** ** ***** ** ** ***

SbMATE AAGCACACTGCCGCTGCCGGCGCGGACCCGGAGAAGCAGCAGCAGCCAGCTGATGAAGAA

oSbMATE aagcatacggccgcggcaggcgctgatccggaaaagcagcagcagcctgccgacgaggag

***** ** ***** ** ***** ** ***** ************** ** ** ** **

SbMATE GCCGCCAAGAACGGCGGCGAGGGATGCGCCCCTGCCGTCGTCGCCGGCCGGAGTAGCGGC

oSbMATE gcggccaagaacggcggggaaggatgtgctcccgcagttgtggccggtcgttccagcggc

** ************** ** ***** ** ** ** ** ** ***** ** ******

SbMATE AAGAAATCAGGGAACAGGAGGTTCGTGCCGTCCGTGACGTCGGCACTGATCGTGGGCGCG

oSbMATE aagaagagtgggaatcgccggttcgtcccatctgtgacctcagcgctgatcgttggcgct

***** ***** * ******* ** ** ***** ** ** ******** *****

SbMATE CTCCTGGGGCTGTTCCAGACCGTCTTCCTCGTCGCCGCCGGGAAGCCGCTGCTGCGCCTC

oSbMATE ctccttggtctgttccagaccgtctttctcgtggccgcgggcaagccgttgctgaggctt

***** ** ***************** ***** ***** ** ****** ***** * **

SbMATE ATGGGCGTCAAGCCGGGTTCGCCCATGGTGATGCCCGCGCTGCGCTACCTGACGCTGCGC

oSbMATE atgggagttaagcctgggagccccatggtgatgccagcactcagatatctgactctccgc

***** ** ***** ** ************** ** ** * ** ***** ** ***

SbMATE GCGCTTGGCGCCCCGGCCGTGCTGCTGTCTCTCGCCATGCAAGGAGTCTTCCGTGGGTTC

oSbMATE gccttgggcgctccggcggtccttctgtccctcgccatgcaaggggtgttcaggggcttc

** * ***** ***** ** ** ***** ************** ** *** * ** ***

SbMATE AAGGACGCCAAGACGCCCTTATACGCCATCGTGGCCGGCGACGCGGCGAACATTGTGCTG

oSbMATE aaggatgctaaaacacctctgtacgcaattgtcgccggtgacgcggccaacatcgttttg

***** ** ** ** ** * ***** ** ** ***** ******** ***** ** **

SbMATE GATCCGATCCTGATATTTGGCTGCCGCCTGGGCGTGATCGGCGCAGCCATTGCCCATGTT

oSbMATE gaccccatcctcatctttggatgccgtcttggcgtgattggggctgcaattgcgcacgtc

** ** ***** ** ***** ***** ** ******** ** ** ** ***** ** **

SbMATE CTTTCCCAGTACCTGATAACGCTGATAATGCTGAGCAAGCTGGTGAGGAAGGTCGATGTC

oSbMATE ctgtcgcagtatctcatcacgctgatcatgcttagtaagttggtgcgcaaagttgatgtc

** ** ***** ** ** ******** ***** ** *** ***** * ** ** ******

SbMATE GTCCCGCCCAGCCTGAAATGCCTCAAATTCCGGCGCTTCCTCGGATGCGGATTCCTTCTG

oSbMATE gtgccaccgtccctcaagtgcctgaagttcaggcggttcctcggctgcggttttcttctg

** ** ** *** ** ***** ** *** **** ******** ***** ** ******

SbMATE CTGGCACGGGTGGTGGCCGTGACGTTCTGCGTGACGCTGGCGGCGTCGCTGGCTGCTCGC

oSbMATE ttggccagagtcgtggctgttaccttctgtgtcactctcgcggccagcctggcagcccgc

**** * ** ***** ** ** ***** ** ** ** ***** ***** ** ***

SbMATE CACGGGCCGACCGCCATGGCCGCCTTCCAGATCTGCACCCAGGTCTGGCTGGCCACGTCC

oSbMATE catggacctacagcgatggctgccttccagatttgcacgcaagtgtggcttgcaacctct

** ** ** ** ** ***** *********** ***** ** ** ***** ** ** **

SbMATE CTCCTCGCCGACGGGCTCGCCGTCGCCGGCCAGGCCATGATCGCGAGCGCCTTCGCCAAG

oSbMATE ctcctggcggacggcttggctgttgccgggcaggcgatgatcgcatcagcctttgctaag

***** ** ***** * ** ** ***** ***** ******** ***** ** ***

SbMATE GAGGACCGCTACAAGGTGGCCGCCACCGCCGCGCGCGTCCTGCAGCTCGGCGTCGTCCTG

oSbMATE gaggatcgttacaaagtggccgcgaccgctgcaagggtcctccagctgggcgtggtcctt

***** ** ***** ******** ***** ** * ***** ***** ***** *****

SbMATE GGCGCCGCCCTCACGGCGCTCCTCGGACTCGGGCTGCAGTTCGGAGCCGGCGTCTTCACC

oSbMATE ggtgccgcgctcactgccctgttgggactcggcctgcaattcggggctggcgttttcaca

** ***** ***** ** ** * ******** ***** ***** ** ***** *****

SbMATE AGCGACGCCGCCGTCATCAAGACCATCCGGAAGGGCGTTCCGTTCGTCGCCGGCACGCAG

oSbMATE tcggacgcagcggtgatcaagacgattcggaagggtgtcccctttgtggccggcacccag

***** ** ** ******** ** ******** ** ** ** ** ******** ***

SbMATE ACGCTCAACACGCTAGCCTTCGTCTTCGACGGCATCAACTTCGGCGCGTCGGACTACGCC

oSbMATE actcttaatacactcgctttcgttttcgatggaatcaactttggggcctccgactacgcg

** ** ** ** ** ** ***** ***** ** ******** ** ** ** ********

SbMATE TTCTCTGCCTACTCCATGATCGGCGTGGCGGCTGTCAGCATCCCGTCGCTCATCTTCCTC

oSbMATE ttcagcgcatattctatgattggcgtcgccgctgtgagtatcccatcactgatcttcttg

*** ** ** ** ***** ***** ** ***** ** ***** ** ** ****** *

SbMATE TCGTCGCACGGCGGCTTCGTCGGCATCTGGGTAGCCCTCACCATCTACATGGGCGTCAGG

oSbMATE tcgtcccacggcggttttgttggaatttgggtcgcgcttacgatctacatgggcgtgaga

***** ******** ** ** ** ** ***** ** ** ** ************** **

SbMATE GCCCTTGCCAGCACCTGGAGGATGGCAGCAGCCCAGGGGCCATGGAAGTTTCTTCGGCAG

oSbMATE gccctcgcaagcacctggcgcatggctgcggcccaagggccgtggaagttcctgaggcag

***** ** ********* * ***** ** ***** ***** ******** ** *****

SbMATE TGA

oSbMATE tga

***

**Supplementary Fig. 4.** Alignment of the nucleotides sequences. Nucleotides alignment of optimized (*oSb*MATE) and original *Sorghum bicolor* MATE **(***Sb*MATE **-** SbMATE03g043890**)** sequences generated by Geneious software (Kearse et al., 2012). (*) Symbols under the alignments indicate identical.
