# Supplementary material for: Enhanced aluminum tolerance in sugarcane: evaluation of SbMATE overexpression and genome-wide identification of ALMTs in Saccharum spp
Source: BMC Plant Biol. 2021 Jun 29;21:300. doi: 10.1186/s12870-021-02975-x (PMC8240408; doi:10.1186/s12870-021-02975-x)
Supplement: Supplementary file 5 — Additional file 5 Supplementary Fig. 5. Alignment of the deduced amino acid sequences. Geneious software (Kearse et al., 2012) alignment of the deduced amino acid sequences of optimized (oSbMATE) and original Sorghum bicolor MATE (SbMATE - SbMATE03g043890) sequences. (*) Symbols under the alignments indicate identical. [file 12870_2021_2975_MOESM5_ESM.doc]

SbMATE MEEHRSPAHAKPEAEQPPQQQVPAAMAVAVAVDVAAPAALQNSTAAPAENGDVAAAGAAE

oSbMATE MEEHRSPAHAKPEAEQPPQQQVPAAMAVAVAVDVAAPAALQNSTAAPAENGDVAAAGAAE

************************************************************

SbMATE NGTAASAANGDGGGSELLGGPRWTGLHLFVMNIRSVFKLDELGAEVLGIAVPASLALTAD

oSbMATE NGTAASAANGDGGGSELLGGPRWTGLHLFVMNIRSVFKLDELGAEVLGIAVPASLALTAD

************************************************************

SbMATE PLASLIDTAFIGRLGSVEIAAVGVAIAVFNQVMKVCIYPLVSVTTSFVAEEDAVLSKGGA

oSbMATE PLASLIDTAFIGRLGSVEIAAVGVAIAVFNQVMKVCIYPLVSVTTSFVAEEDAVLSKGGA

************************************************************

SbMATE KVIDNGEEEEELEAGQVGPEKHTAAAGADPEKQQQPADEEAAKNGGEGCAPAVVAGRSSG

oSbMATE KVIDNGEEEEELEAGQVGPEKHTAAAGADPEKQQQPADEEAAKNGGEGCAPAVVAGRSSG

************************************************************

SbMATE KKSGNRRFVPSVTSALIVGALLGLFQTVFLVAAGKPLLRLMGVKPGSPMVMPALRYLTLR

oSbMATE KKSGNRRFVPSVTSALIVGALLGLFQTVFLVAAGKPLLRLMGVKPGSPMVMPALRYLTLR

************************************************************

SbMATE ALGAPAVLLSLAMQGVFRGFKDAKTPLYAIVAGDAANIVLDPILIFGCRLGVIGAAIAHV

oSbMATE ALGAPAVLLSLAMQGVFRGFKDAKTPLYAIVAGDAANIVLDPILIFGCRLGVIGAAIAHV

************************************************************

SbMATE LSQYLITLIMLSKLVRKVDVVPPSLKCLKFRRFLGCGFLLLARVVAVTFCVTLAASLAAR

oSbMATE LSQYLITLIMLSKLVRKVDVVPPSLKCLKFRRFLGCGFLLLARVVAVTFCVTLAASLAAR

************************************************************

SbMATE HGPTAMAAFQICTQVWLATSLLADGLAVAGQAMIASAFAKEDRYKVAATAARVLQLGVVL

oSbMATE HGPTAMAAFQICTQVWLATSLLADGLAVAGQAMIASAFAKEDRYKVAATAARVLQLGVVL

************************************************************

SbMATE GAALTALLGLGLQFGAGVFTSDAAVIKTIRKGVPFVAGTQTLNTLAFVFDGINFGASDYA

oSbMATE GAALTALLGLGLQFGAGVFTSDAAVIKTIRKGVPFVAGTQTLNTLAFVFDGINFGASDYA

************************************************************

SbMATE FSAYSMIGVAAVSIPSLIFLSSHGGFVGIWVALTIYMGVRALASTWRMAAAQGPWKFLRQ

oSbMATE FSAYSMIGVAAVSIPSLIFLSSHGGFVGIWVALTIYMGVRALASTWRMAAAQGPWKFLRQ

************************************************************

**Supplementary Fig. 5.** Alignment of the deduced amino acid sequences. Geneious software (Kearse et al., 2012**)** alignment of the deduced amino acid sequences of optimized (o*Sb*MATE) and original *Sorghum bicolor* MATE **(***Sb*MATE **-** SbMATE03g043890**)** sequences. (*) Symbols under the alignments indicate identical.
