# Supplementary material for: Enhanced aluminum tolerance in sugarcane: evaluation of SbMATE overexpression and genome-wide identification of ALMTs in Saccharum spp
Source: BMC Plant Biol. 2021 Jun 29;21:300. doi: 10.1186/s12870-021-02975-x (PMC8240408; doi:10.1186/s12870-021-02975-x)
Supplement: Supplementary file 6 — Additional file 6 Supplementary Table 1 Sequence of the pair of primers used for PCR and qPCR analysis. [file 12870_2021_2975_MOESM6_ESM.doc]

**Supplementary Table 1** Sequence of the pair of primers used for PCR and qPCR analysis.

| Oligo Name | | Sequence (5’ → 3’) | Amplicon  Size | Annealing TºC |
| --- | --- | --- | --- | --- |
| o*Sb*MATE | *F^*^* | CATTTGTGGCAGAGGAGGAC | 301 pb | 59 |
|  | *R^**^* | TGGAACAGACCAAGGAGAGC |  |  |
| *Zm*Ubi1 | *F^*^* | CGAGTAGATAATGCCAGCCT | 456 pb | 58 |
|  | *R^**^* | GACGAGCGGCGTACCTTGAA |  |  |
| *bar* | *F^*^* | TGTTTATCGGCACTTTGCAT | 262 pb | 54 |
|  | *R^**^* | GATGTTGGCGACCTCGTATT |  |  |
| *So*GAPDHq | *F^*^* | CACGGCCACTGGAAGCA | 80 pb | 60 |
|  | *R^**^* | TCCTCAGGGTTCCTGA |  |  |
| *So*EF1-αq | *F^*^* | TTTCACACTTGGAGTGAAGCAGAT |  |  |
|  | *R^**^* | GACTTCCTTCACAATCTCATCATAA |  |  |
| *So*MATEq | *F^*^* | GCCTGTATCTGGTCCTGATTTG |  |  |
|  | *R^**^* | CTCTTCTTGCACCCTTGGTTAG |  |  |
| *So*STOP1q | *F^*^* | GCGAGGAGCATGGTGTATAG |  |  |
|  | *R^**^* | AGTTCAGCGGCGAGAAATAA |  |  |
| *So*STAR1q | *F^*^* | CCATCTCGACGCAGAACAT |  |  |
|  | *R^**^* | CTTCACGCTGTGGGAGAC |  |  |
| *So*NRAT1q | *F^*^* | CCAGCTTCTCTGGGTGATTT |  |  |
|  | *R^**^* | AAGATGCTTCCCTGTCTTCAC |  |  |
| *So*CYSq | *F^*^* | CTGTCCCAGCTCATGTCTATAAG |  |  |
|  | *R^**^* | TCTCAACCTGAAGAGCCATTAC |  |  |
| *So*MDHq | *F^*^* | CCAGATATCGTTGAGTGCTCTTT |  |  |
|  | *R^**^* | GCACTTCCTCAACTCCATTCT |  |  |
| *So*FUMq | *F^*^* | GAGAGCAGTGGTGCAGTAAA |  |  |
|  | *R^**^* | AGTTCACCAAGACCACATCG |  |  |
| *So*ALMT2q | *F^*^* | CCCTCACAAGAACTGAGATGAG |  |  |
|  | *R^**^* | GCCTCTTGCTGCTTTGATTG |  |  |
| *So*ALMT4q | *F^*^* | GCTCGTCTCCGTCTTCTACTA |  |  |
|  | *R^**^* | TCGACTCACCGACAGTGTAT |  |  |
| *So*ALMT5q | *F^*^* | CCTTGGCTACATTTGCATCAC |  |  |
|  | *R^**^* | GCCTTCTCACTCAACTCTTCA |  |  |

**Forward*; ***Reverse*

**Supplementary Table S2.** **Continued …**

| Oligo Name | | Sequence (5’ → 3’) | Amplicon  Size | Annealing TºC |
| --- | --- | --- | --- | --- |
| *So*ALMT7q | *F^*^* | GCTGGTGATAGAGGAAGTGAAG | 80 pb | 60 |
|  | *R^**^* | CTACTGAGATCAACGACCAGATG |  |  |
| *So*ALMT9q | *F^*^* | CTGGGTACAACACTGGGAAAT |  |  |
|  | *R^**^* | ACTGACAGCAGCACCAATAG |  |  |
| *So*ALMT11q | *F^*^* | CAAGGATGAATCACCCGAGAA |  |  |
|  | *R^**^* | AATCGTATCTGCCGAGGAATG |  |  |

**Forward*; ***Reverse*
