# Supplementary material for: Enhanced aluminum tolerance in sugarcane: evaluation of SbMATE overexpression and genome-wide identification of ALMTs in Saccharum spp
Source: BMC Plant Biol. 2021 Jun 29;21:300. doi: 10.1186/s12870-021-02975-x (PMC8240408; doi:10.1186/s12870-021-02975-x)
Supplement: Supplementary file 7 — Additional file 7 Supplementary Fig. 6 (a) Hydroponics system of sugarcane in greenhouse. (b) Sugarcane transgenic plants SbMATE and NT plants in the hydroponic system, before aluminum treatment. [file 12870_2021_2975_MOESM7_ESM.doc]

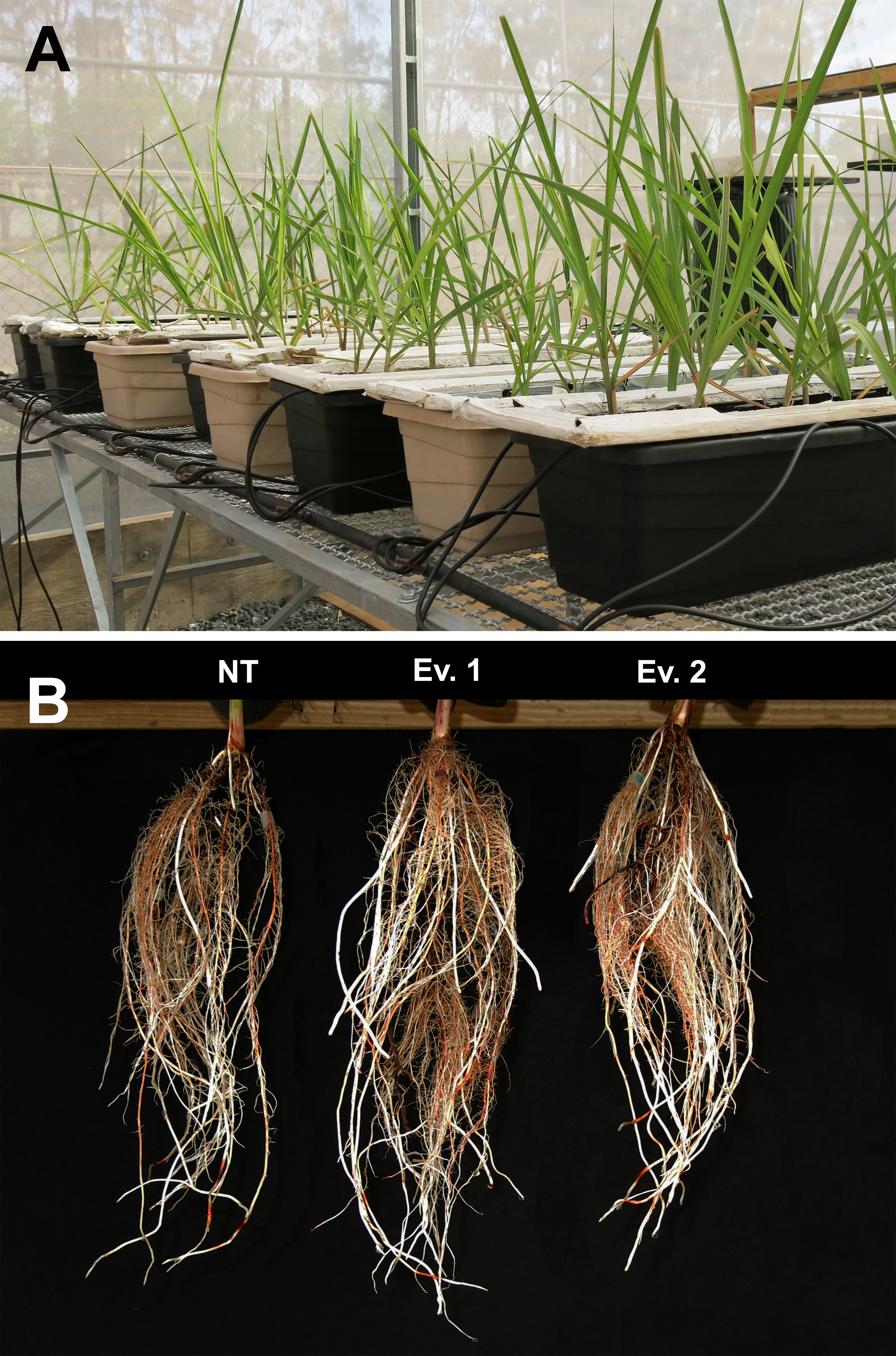


**Supplementary Fig. 6 (a)** Hydroponics system of sugarcane in greenhouse. **(b)** Sugarcane transgenic plants *Sb*MATE and NT plants in the hydroponic system, before aluminum treatment.
